# Enhancing theranostic potential of anti-mesothelin sdAb through site-specific labeling at a unique conserved lysine by molecular engineering

**DOI:** 10.1186/s41181-025-00340-z

**Published:** 2025-04-28

**Authors:** Émilien N’Guessan, Florian Raes, Mitra Ahmadi, Sandrine Bacot, Laurent Dumas, Julien Leenhardt, Marlène Debiossat, Clémence André, Jean-Luc Lenormand, Catherine Ghezzi, Daniel Fagret, Charlotte Lombardi, Alexis Broisat

**Affiliations:** 1 Univ. Grenoble Alpes, INSERM U1039, LRB, Grenoble, France; 2 Department of Nuclear Medicine, Univ. Grenoble Alpes, CHU Grenoble Alpes, Grenoble, France; 3 Univ. Grenoble Alpes, CNRS U5525, TIMC-Tree, La Tronche, France

**Keywords:** Site-specific radiolabeling, sdAb, Mesothelin, Theranostic, Kidney retention

## Abstract

**Background:**

Mesothelin is a 40 kDa glycoprotein overexpressed in several cancers, including triple-negative breast cancer (TNBC). The anti-mesothelin single-domain antibody (sdAb, or nanobody) A1 can serve as a radio-theranostic agent, but random DOTA conjugation on lysines yields heterogeneous products.

**Results:**

We reengineered A1-His by directed mutagenesis to produce four single-lysine variants (A1K1-His, A1K2-His, A1K3-His, and A1K4-His). Each was site-specifically conjugated with p-SCN-Bn-DOTA, radiolabeled with ^68^Ga, and evaluated by PET imaging in mice bearing HCC70 TNBC xenografts, followed by ex vivo biodistribution at 1 h post-injection. All mutants were successfully produced and site-specifically conjugated. A1K1-His showed lower conjugation efficiency and increased liver/spleen retention, whereas A1K3-His exhibited reduced stability. A1K2-His and A1K4-His performed best overall. Removing the His-tag and administering gelofusin further lowered renal uptake. Notably, A1K2 displayed tumor-to-kidney and tumor-to-liver ratios 2.4 and 1.9 times higher, respectively, than A1K4 (*p* < 0.01).

**Conclusions:**

For the first time, site-specific DOTA conjugation using sdAb derivatives containing a single lysine was achieved, avoiding the production of mixed final compounds. These findings identify ^68^Ga-DOTA-A1K2 as the leading candidate for mesothelin-expressing tumor imaging with minimal off-target uptake. Ongoing studies will assess its therapeutic utility with ^177^Lu-DOTA-A1K2. Since these four lysines are conserved in many sdAbs, this strategy may be broadly applicable for site-specific sdAb labeling.

**Supplementary Information:**

The online version contains supplementary material available at 10.1186/s41181-025-00340-z.

## Background

Single-domain antibodies (sdAbs), also known as VHHs or nanobodies, correspond to the antigen-binding domain of heavy-chain-only antibodies found in Camelidae. sdAb-based imaging agents are widely employed due to their advantageous properties (Jin et al. 2023). One of their main advantages lies in the combination of very high specificity, in the nano- to picomolar range, with rapid blood clearance, allowing the acquisition of images with elevated target-to-background ratios within an hour post-injection (Harmand et al. 2021). They can easily be generated against a wide range of extra cellular targets, and numerous sdAbs have already been validated for nuclear SPECT and PET imaging (Harmand et al. 2021). Tumor imaging, in particular, has largely benefited from the development of sdAb-based radiotracers. Indeed, numerous membrane proteins overexpressed in solid or hematological tumors have been successfully imaged using sdAbs. Among them, some have reached clinical trials, including anti-human epidermal growth factor receptor 2 (HER2) (Gondry et al. 2024), anti-programmed death-ligand 1 (PDL-1) (Zhang et al. 2024) and anti-macrophage mannose receptor (MMR) sdAbs (Gondry et al. 2023), thereby confirming the potency of this class of imaging agents for clinical use. In oncology, sdAb-based imaging agents can be employed either as prognostic markers that provide insight into parameters such as tumor aggressiveness (Montemagno et al. 1039), as companion diagnostics for targeted therapies directed against the same protein (Qi et al. 2024), and as radio-theranostic tools that can also be employed for therapy following labeling with beta- or alpha emitters (Lecocq et al. 2019).

Like for any other nuclear imaging agent, the biodistribution profile is a key parameter to optimize when developing a sdAb-based radiotracer. Minimal uptake in off-target tissues is essential for tumor detection with high sensitivity, especially in metastatic diseases. It is also of tremendous importance in terms of dosimetry, particularly when the sdAb is intended to be used as a theranostic tool. SdAbs are known to rapidly accumulate in tumors while clearing from the circulation and off-target organs. However, there is still a need to improve their pharmacokinetics in order to enhance their potency. Most efforts have been focused on reducing kidney retention, which is a major drawback for their use as radio-theranostic tools since its represent their major elimination pathway (Tchouate Gainkam et al. 2011). This has been addressed by co-injecting competitors of kidney re-uptake such as amino acids or gelofusin, by removing tags located on C-termini regions (D’Huyvetter et al. 2014), or by modifying the radiochemistry approach since the choice of chelator can strikingly impact the retention of the radioelement within the kidney cortex (Baudhuin et al. 2021; D’Huyvetter et al. 2012). Another strategy is to use residualizing prosthetic groups, such as N-succinimidyl-4-guanidinomethyl-3-[I-iodobenzoate] (SGMIB), that are hydrophilic and positively charged upon proteolytic degradation (Dewulf et al. 2024).

Radiometals, however, remain the most widely used isotopes for SPECT and PET imaging with sdAbs. Among them, ^68^Ga labeling is often used for PET imaging since it allows rapid and efficient labeling of sdAbs and is widely available through generators, including for clinical use (Banerjee and Pomper 2013). ^68^Ga labeling is usually performed following the addition of a bifunctional chelator to side-chain lysines. While NOTA is the most straightforward choice for PET imaging with ^68^Ga, allowing radiolabeling at room temperature, DOTA offers the advantage of also complexing beta- emitters such as lutetium-177 or terbium-161 for theranostic purposes (Stasiuk and Long 2013; Busslinger et al. 2024).

Coupling NOTA or DOTA chelators to side-chain lysines has been well validated, including in clinical practice with anti-HER2 and anti-MMR sdAbs for example. However, one potential drawback of this approach is the generated mixture of sdAbs bearing a variable number of chelators at different locations since four conserved lysines are usually found within the framework (Hirao et al. 2023; Mitchell and Colwell 2018). Moreover, the radiolabeling also results in a mixture in terms of the number of ^68^Ga per sdAb, which can lead to significant differences in properties between variants. Recently, Baudhuin et al. investigated the impact of the number of chelators per sdAb on their pharmacokinetics, using a highly resolutive anion exchange chromatography column to separate them prior to radiolabeling (Baudhuin et al. 2021). While the conjugation degree did not induce major changes in the biodistribution of the sdAbs, a few modest but significant changes were observed for the liver and kidneys. However, while this study successfully evaluated the impact of the number of chelators per sdAb, it did not address the impact of their location within the framework, which could potentially also affect chelator conjugation efficiency, radiolabeling, stability, and pharmacokinetics.

One solution to avoid such mixtures consists of fixing only a single chelator per sdAb, for example by using a sortase approach (Basuli et al. 2024). However, sortase methods require the addition of extra enzymes and substrate peptide sequences, complicating production and purification processes. Here, we investigated an innovative straightforward method involving directed mutagenesis on the sdAb sequence to replace three out of four lysines by arginines, thus keeping a single lysine available for the bifunctional chelator’s conjugation, leading to site-specific ^68^Ga labeling on a side-chain lysine. This approach enables to skip the use of an additional step such as sortase and thus greatly facilitates the production and purification of the final sdAb. Importantly, by avoiding modifications at the C-terminal site of the sdAb, we maintained its natural clearance properties, and so prevent potential increases in renal retention associated with the presence of polar Aa on C-terminus (Li et al. 2023).

For this proof of concept study, we used the anti-human mesothelin sdAb A1 previously validated by our group for SPECT imaging of triple-negative breast cancer and pancreatic ductal adenocarcinoma following radiolabeling with technetium-99 m (Montemagno et al. 2018, 1531). Mesothelin physiologic expression is restricted to mesothelial cells, but is overexpressed in various cancers with poor prognosis that yet lack targeted therapies (Weidemann et al. 2021; Faust et al. 2022). The thermal stability of sdAb allowed the use of DOTA as the chelator. For each of the four mutants of A1, we investigated the impact of the remaining lysine site (named K1 to K4) on the sdAb's affinity, on the efficiency of coupling with the bifunctional chelator, on the radiolabeling rate and stability, and on the in vivo biodistribution. In addition, previously validated approaches to reduce kidney retention was also employed to further improve the pharmacokinetics of the most potent mutants.

## Methods

### Three-dimensional structure prediction

AlphaFold v3 predictions were run on the open-access AlphaFold server. Residue scores and sequences can be found in the supplementary materials (Supplementary Figure S1). The predicted structure of the sdAb A1 was further validated by comparing it to other sdAbs as well as to that previously obtained by our group using crystallography (Supplementary Figure S1-C). The Chimera extensible molecular modeling software was used for structural comparison and visualization. Hydrogen bonds between A1 and hMSLN were investigated using ChimeraX, depicting hydrogen bonds up to 3.5 Å (Goddard et al. 2018; Pettersen et al. 2021).

### Production of A1-His, A1Kn-His, A1, and A1Kn

A1-His and its mutants, A1Kn-His, were re-cloned into the pET-15b expression vector incorporating a hexahistidine tag. Moreover, the cleavage site (ENLYFQ) recognized by tobacco etch virus (TEV) protease was added between A1Kn and the hexahistidine tag (Swale et al. 2016). Transformation was performed using SHuffle T7 Express Competent *E. coli* C3029J (New England Biolabs), and expression of sdAbs was induced by adding 1 mM isopropyl-β-d-thiogalactoside (IPTG) with incubation at 37 °C overnight. Soluble extracts containing sdAbs were obtained through lysozyme treatment and sonication, followed by centrifugation. To produce sdAbs without the His-tag, the proteins underwent TEV protease cleavage at a 1:1 (w/w) ratio during an overnight dialysis step using a 3.5 kDa molecular weight cutoff membrane (Spectrum™ Spectra/Por™ 7) in Tris/NaCl buffer. Cleavage of the His-tag was confirmed through Western blotting using an anti-His horseradish peroxidase conjugated antibody. Subsequent purification of both His-tagged and His-tag-cleaved sdAbs involved immobilized metal affinity chromatography (IMAC) on Ni–NTA resin (Sigma-Aldrich) in Tris/NaCl buffer with 20 nM to 300 nM imidazole and gel filtration on a Superdex 75 HR 16/60 column (Cytiva) in Tris/NaCl buffer.

### Analysis of the affinity and thermal stability

The affinity of sdAbs for recombinant human mesothelin (PeproTech) was determined using surface plasmon resonance (SPR) analysis on a Biacore T200 apparatus. Recombinant mesothelin was immobilized on a CM5 sensor chip (Cytiva), and sdAbs were flowed over the immobilized protein (in a twofold dilution series from 100 nM to 0.4 nM). Affinity constants were determined using a 1:1 kinetic standard association model fit (BIA T200 control software).

Thermal stability analysis was conducted using circular dichroism (CD) on a Jasco spectropolarimeter. Samples were diluted in PBS:water (3:5) at a concentration of 0.4 mg/mL CD spectra were recorded from 205 to 260 nm over a temperature range of 25–80 °C.

### DOTA conjugation

Various conjugation conditions with DOTA were evaluated, and then the optimal conditions were applied to all other derivatives. First, sdAbs (in Tris-NaCl buffer, pH 7.4) were buffer-exchanged into 0.5 M sodium carbonate buffer, pH 9.0 (sodium carbonate anhydrous–sodium hydrogen carbonate; Sigma-Aldrich) using PD-10 size-exclusion disposable columns (GE Healthcare). A 50-fold molar excess of p-SCN-Bn-DOTA (3 mg/mL) was added to the protein solution (1.5 mg/mL), and the pH was adjusted to 9.0. The mixtures were incubated for 3 h at 25 °C under stirring at 300 rpm.

After each conjugation reaction, the pH was adjusted to 7–7.5, and the solution was purified by size exclusion chromatography (SEC) using semi-preparative high-pressure liquid chromatography (HPLC; Shimadzu). SEC was performed on a Superdex 75 10/300 column (Cytiva) with an isocratic mobile phase of 0.1 M ammonium acetate (Trace metal) (pH 6.8) at a flow rate of 0.75 mL/min for 40 min to separate the conjugated sdAb from excess DOTA. UV detection was set at 225 nm and 277 nm for DOTA and sdAb detection, respectively. The purified conjugates were then concentrated to 1 mg/mL using Amicon Ultra-15 filters (Ultracel® 3 K). All samples were analyzed by MALDI-TOF MS using an Autoflex Speed mass spectrometer (Bruker Daltonics) in linear positive mode, with samples prepared using the sinapinic acid double-layer method. After purification and analysis, each DOTA-conjugated sdAb sample was stored at –80 °C.

### Radiolabeling and radiochemical purity

7–8 nmol of each DOTA-conjugated sdAb (100 µg in 0.1 M ammonium acetate) were mixed with 0.030 mL of 1 M ammonium acetate buffer to achieve a final pH of 3.5. Subsequently, 0.5 mL (150–185 MBq) of ^68^Ge/^68^Ga generator eluate (IRE ELiT®, Belgium) was added. The mixture was heated at 60 °C for 15 min under continuous stirring. After radiolabeling, each sdAb solution was purified by gel filtration on a NAP-5 column (Sephadex™ G-25 DNA Grade Gel; GE Healthcare) equilibrated with PBS (Sigma-Aldrich, Cat # D8662) and then filtered through a 0.22 µm filter (Millex, Millipore).

Radiochemical purity (RCP), was assessed using reversed-phase high-performance liquid chromatography (RP-HPLC). The analytical HPLC system (Shimadzu) was equipped with a radiodetector (Elysia-Raytest) and a UV detector (Shimadzu), utilizing a C4 reverse-phase column (Symmetry300™, 5 μm, 150 × 4.6 mm; Waters). The elution was performed with solvents A (0.1% trifluoroacetic acid (TFA) in water) and B (0.1% TFA in acetonitrile) at a flow rate of 1 mL/min under the following gradient conditions: 0–1 min, 5% B; 1–5 min, linear gradient from 5 to 90% B; 5–7 min, 90% B; 7–10 min, returning to initial conditions; and 10–12 min, 5% B. The radiochemical purity of each radiolabeled sdAb was determined by integrating the radioactivity peaks corresponding to the sdAb and any free radionuclide or impurities.

### Cell lines and culture conditions

The HCC70 cell line, obtained from the American Type Culture Collection, was cultured in RPMI 1640 medium (Sigma-Aldrich) supplemented with 10% fetal bovine serum (FBS; Sigma-Aldrich) and 1% penicillin–streptomycin (Sigma-Aldrich). Cells were incubated at 37 °C with 5% CO₂.

### Animal models

Approval for all procedures was obtained from the animal care and ethics committee of Grenoble Alpes University and the French Ministry (APAFIS#19,480–2019022616164184 v4). Mice had access to rodent chow and water ad libitum. They were maintained on a 12-h light/dark cycle, with temperature between 20 and 24 °C and relative humidity of 40–60%. A one-week acclimatization period was observed prior to cells implantation.

Female athymic nude mice (Charles River, France; Janvier, France) at 5 weeks of age were subcutaneously xenografted in the left posterior upper leg with HCC70 cells (3.5 × 10^6^ cells, total n = 92, in a 1:1 (v/v) PBS/Matrigel (Corning) mixture. Tumor growth was monitored using caliper 2 to 3 times a week, allowing tumors to grow for 3–4 weeks to reach approximately 200–400 mm^3^.

### PET-CT imaging

PET/CT acquisitions were performed 1 h after intravenous injection via the tail vein of 2–9 MBq of the ^68^Ga -DOTA-sdAbs. Stratified randomization ensured similar weights and tumor volumes between groups. Details of group sizes, mean weights, mean tumor volumes, and mean injected activities for each group can be found in the supplementary data (tables S.1, 4, 7, 10 and 13). For some groups, an injection of 100 µL of 4% Gelofusin® was administered 1–3 min before the radiotracer injection to reduce kidney retention. Signal specificity was assessed either by in vivo competition or in vivo displacement assay, using a 100-fold excess an unlabelled competitor (A1-His) or unlabelled unspecific sdAb (R3B23-His (Lemaire et al. 2014)) that was injected simultaneously or 60 min after the ^68^Ga -labeled sdAb, respectively. PET/CT acquisitions were then started immediately and continued for 40 min. PET/CT acquisitions were performed using dedicated system (nanoPET/CT Mediso). Images were corrected for decay and normalized to the injected dose.

### Biodistribution studies

Two hours after injection and immediately after image acquisition, the anesthetized mice were euthanized by cervical dislocation, and tumors were harvested along with other organs. Tissues were weighed, and tracer activity was determined with a γ-counter (Wizard2, PerkinElmer). The results were corrected for decay, injected dose, and organ weight, and were expressed as a percent injected dose per gram (%ID/g). Tumor-to-liver, tumor-to-kidney, and tumor-to-blood activity ratios were computed.

### Statistical analysis

Mean values ± standard deviation (SD) were compared using nonparametric, unpaired tests: the Mann–Whitney U test for comparison of two groups and the Kruskal–Wallis test with Dunn’s correction for comparison of more than two groups. *P* values of 0.05 or less were considered significant. Analyses were conducted with GraphPad Prism version 8.0.2.

## Results

### Three-dimensional structure and in silico results

**Fig. 1:**
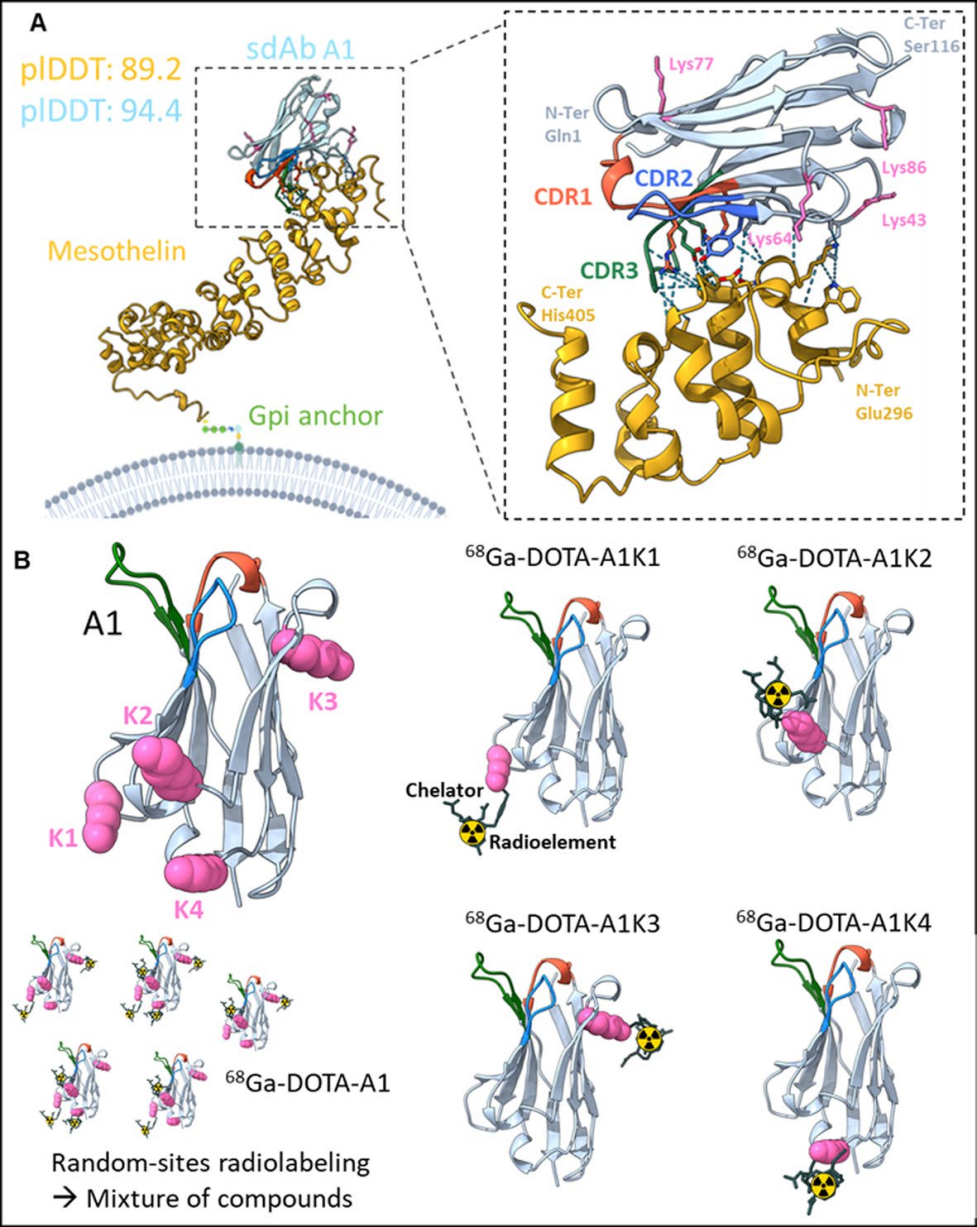
3D structures of the sdAbs A1 and its mutants. (**A**) Schematic representation of sdAb A1, and its interaction with the membrane protein mesothelin predicted by Alpha Fold v3. Zoom on the hydrogen bonds up to 3.5 Å. (**B**) Illustrative representations of sdAb A1 and its mutants, depicting site-selective radiolabeling. Lys43 correspond to K1, Lys64 to K2, Lys77 to K3 and Lys86 to K4

The three-dimensional structure of the A1 sdAb complexed with mesothelin was modeled using AlphaFold v3, revealing high predicted Local Distance Difference Test (pLDDT) scores for mesothelin (89.2) and A1 (94.4). Superposition of the AlphaFold-generated models of mesothelin and sdAb A1 with their respective crystallographic data (Zhan et al. 2023) confirmed accurate predictions of tertiary structures (supplementary Fig. 1C). K2-3 lysine residues in A1 were found to be positioned away from the complementarity-determining regions (CDRs) and from anti-parallel beta sheets formed by Aa located within the framework downstream of the CDR2, that were found to be involved in the binding to mesothelin. Similarly, K1 (Lys43) only showed a minor interaction with K319 of mesothelin (Fig. 1A). Consequently, mutating these Aa is not expected to interfere with A1 binding capacity, and this finding also suggest that each lysine residue is potentially suitable for DOTA chelation, which is important for subsequent radiolabeling. In silico predictions indicate that substituting lysine by arginine should not alter the overall 3D structure or the interactions between mesothelin and A1, and that the remaining lysine residue should be well accessible for DOTA complexation and radiolabeling. Based on Alpha-fold predictions, all four mutants could therefore potentially enable site-specific conjugation with p-SCN-Bn-DOTA on the remaining lysine, avoiding the mixture of compounds initially obtained with A1 (Fig. 1B).

### Affinity, thermal stability and DOTA coupling

**Fig. 2:**
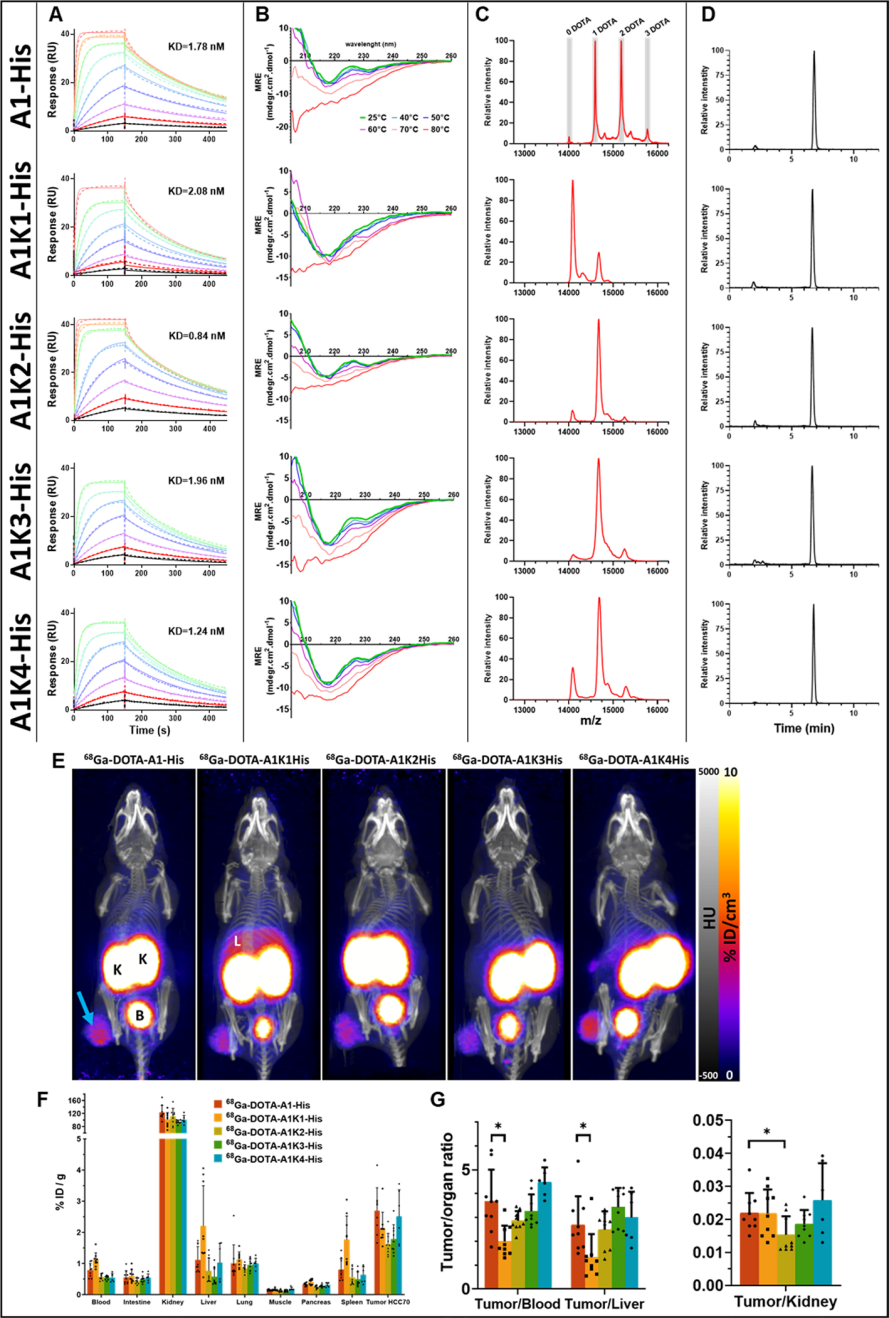
In vitro and in vivo comparisons of the sdAb A1-His, with its 4 mutants A1K1-His, A1K2-His, A1K3-His and A1K4-His. (**A**) Affinity evaluation by SPR for hMSLN, using a twofold dilution series up to 100 nM (red) to 0.4 nM (black). (**B**) Thermal stability evaluation by circular dichroism (CD). CD spectra obtained at different temperatures ranging from 25 to 80 °C. (**C**) MALDI-TOF–MS spectra. (**D**) RP-HPLC radiochromatograms after radiolabeling with ^68^Ga. (**E**) Representative PET/CT images 2 h after intravenous injection of ^68^Ga-DOTA-A1-His or its mutants. Blue arrows indicate HCC70 tumor; K = kidney; B = bladder; L = liver. (n = 9, except A1K4His n = 6). (**F**) Ex vivo biodistribution profile 2 h after intravenous injection. *Significantly different from control sdAb ^68^Ga-DOTA-A1-His (* = *p* < 0.05). (**G**) Tumor-to-blood, tumor-to-liver, and tumor-to-kidney ratios 2 h after injection

All sdAbs, A1-His and its four mutants (A1K1-His, A1K2-His, A1K3-His, and A1K4-His) were successfully cloned into the pET-15b plasmid and expressed in *E. coli Shuffle* cells. Following purification through immobilized metal affinity chromatography (IMAC) and size-exclusion chromatography (FPLC), yields ranged from 6.3 to 11.9 mg of sdAb per liter of lysogeny broth culture medium (Table 1). The affinities of these sdAbs for human recombinant mesothelin (hMSLN) were determined by SPR and found to be comparable, with dissociation constants (KD) between 0.84 and 2.08 nM (Table 1). Affinity data for all sdAbs are depicted in Fig. 2A.

**Table 1:** In vitro characteristics of the sdAbs A1-His and all its mutants A1Kn-His and A1Kn

	Yield production (mg/L culture)	KD (nM)	K_on_ (1/Ms) E + 06	K_off_ (1/s) E-03	DOTA/sdAb	Radiolabelling %RCP t_0_	Stability %RCP t_2h_
A1-His	11,9	1.78	2.07	3.63	1.8 ± 0.32	96,0 ± 3,2	96.0 ± 3.2
A1K1-his	8,8	2.08	3.89	7.23	0.43 ± 0.19	94,3 ± 0,6	92.3 ± 0.6
A1K2-his	10,0	0.84	5.89	4.77	0.87 ± 0.02	94,3 ± 4,7	89.7 ± 3.2
A1K3-his	11,9	1.96	2.89	5.26	0.72 ± 0.35	91,3 ± 6,8	74.0 ± 22.7
A1K4-his	6,3	1.24	4.15	4.83	0.68 ± 0.09	94,0 ± 4,4	89.7 ± 3.5
A1K2	2,9	2.29	3.39	7.61	0.69 ± 0.13	95,0 ± 2,0	92.7 ± 1.5
A1K4	0,9	3,64	3.10	11.21	0.72 ± 0.03	94,3 ± 2,1	93.0 ± 1.0

Given the slow reaction kinetics of DOTA and the high temperatures required for radiolabeling, which may affect sdAb integrity, thermal resistance was assessed using CD spectroscopy. For all sdAbs, 60 °C was selected as the optimal temperature for radiolabeling without compromising structural integrity (Fig. 2B).

Optimal DOTA conjugation conditions were identified as a 50-fold molar excess of p-SCN-Bn-DOTA at pH 9, incubated at 25 °C for 3 h with stirring at 300 rpm. After purification via SEC-HPLC, the conjugation for A1-His yielded 34.0 ± 9% mono-conjugated, 51.0 ± 1% di-conjugated, and 14.3 ± 10% tri-conjugated fractions (Fig. 2C). This resulted in an average of 1.8 ± 0.32 DOTA chelators per sdAb, with less than 1% unconjugated sdAb.

Similar conjugation parameters were applied to each A1Kn-His mutant and the two derivatives without the His-tag (A1K2 and A1K4). Comparable conjugation rates were obtained with A1K2-His, A1K3-His and A1K4-His that were of 0.87 ± 0.02, 0.7 ± 0.35 and 0.68 ± 0.09 DOTA per sdAb, respectively (p = NS). However, a lower conjugation rate (0.43 ± 0.19 DOTA per sdAb) was observed for A1K1-His, that reached statistical significance compared to A1K2-His (*p* < 0.05). (Fig. 2C and Table 1).

### Radiolabeling and stability

RCPs were assessed in triplicate by radio-HPLC immediately and 2 h after labelling and expressed as mean ± SD (Table 1). Prior to purification, all sdAbs demonstrated high initial RCPs, ranging from 91.3 ± 6.8% (^68^Ga -DOTA-A1K3-His) to 96.0 ± 3.2% (^68^Ga -DOTA-A1-His). Stability at 2 h was > 90% for ^68^Ga -DOTA-A1-His and ^68^Ga -DOTA-A1K1-his, while a trend toward a lower RCP was observed for the other mutants and notably for ^68^Ga -DOTA-A1K3-His that diminished to 74.0 ± 22.7% (*p* < 0.05 vs ^68^Ga -DOTA-A1K1-His). Prior to biological studies, all radiolabeled sdAbs were purified, achieving RCPs greater than 95%. (Fig. 2D an Table 1).

### In vivo evaluation of ^68^Ga-DOTA-A1-His and his mutants ^68^Ga-DOTA-A1kn-His

Mice bearing HCC70 xenograft tumors were injected with ^68^Ga -DOTA-A1-His or one of its four mutants. As expected, predominant non-specific uptake was observed in the kidneys, with retention levels ranging between 94.4 ± 5.4% ID/g and 123.3 ± 22.0% ID/g. Tumor uptake was recorded 2 h post-injection, ranging between 1.6 and 2.7%ID/g for ^68^Ga -DOTA-A1-His and its mutants, uptake in other organs are provided in Supplementary Tables (Fig. 2E, F and STable 1–3). Notably, ^68^Ga -DOTA-A1K1-His exhibited different profile of biodistribution than that of the other 3 mutants, resulting on significantly lower tumor-to-blood and tumor-to-liver ratios (*p* < 0.01 vs ^68^Ga -DOTA-A1-His) (Fig. 2G).

### Design, synthesis and characterization of A1K2 and A1K4

**Fig. 3:**
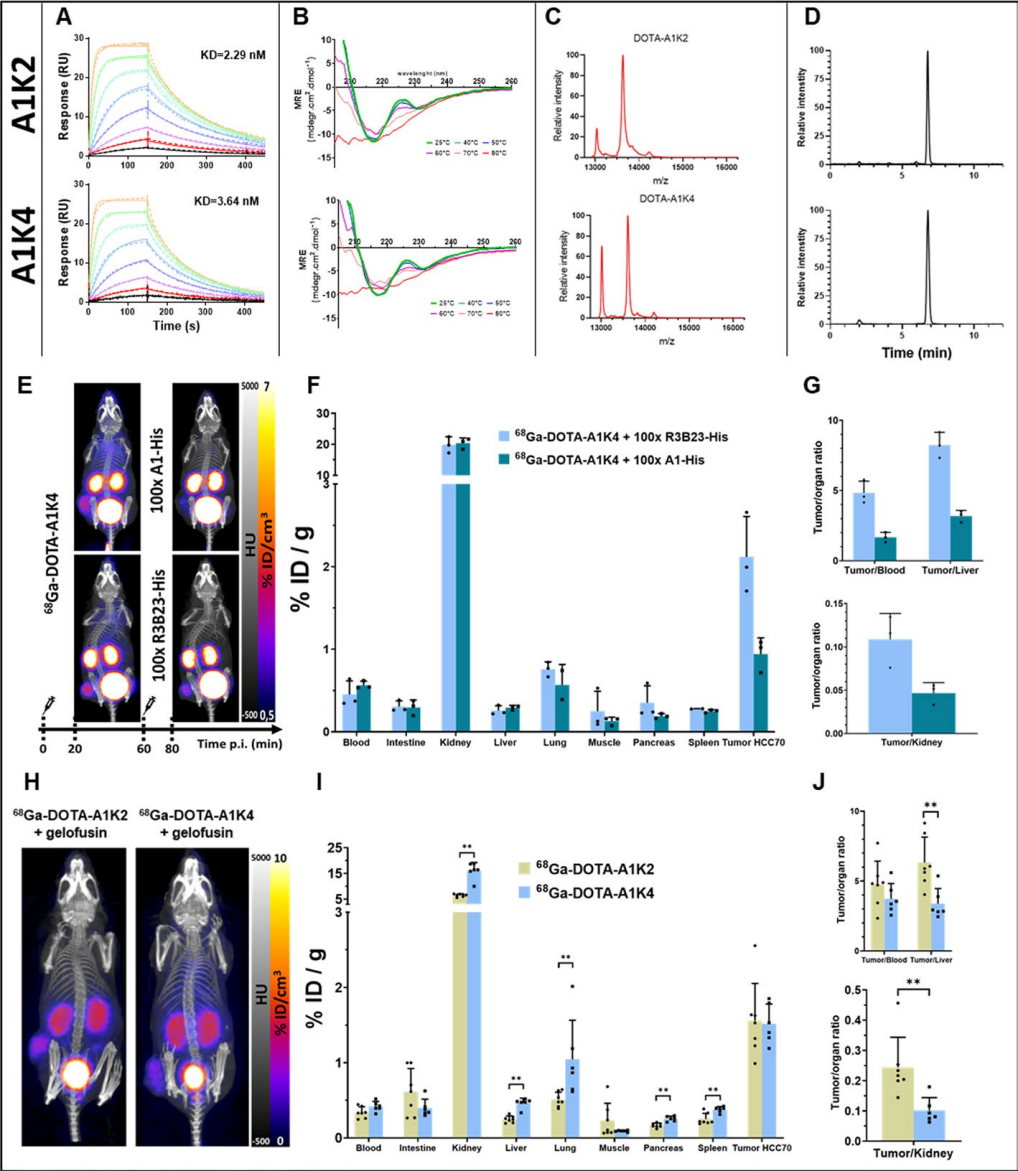
In vitro and in vivo comparison of sdAbs A1K2 and A1K4. (**A**) Affinity evaluation by SPR, using a twofold dilution series from up to 50 nM (orange) to 0.4 nM (black). (**B**) Thermal stability evaluation by circular dichroism (CD). CD spectra obtained at different temperatures ranging from 25 to 80 °C. (**C**) MALDI-TOF–MS spectra. (**D**) RP-HPLC radiochromatograms after radiolabeling of ^68^Ga-DOTA-A1K2 and ^68^Ga-DOTA- A1K4. (**E**) Representative images of the displacement study after intravenous injection. Maximum intensity projection representative of merged PET/CT. (n = 3). **(F**) Ex vivo biodistribution profile, 2 h after intravenous injection. (**G**) Tumor-to-blood, tumor-to-liver and tumor-to-kidney ratios, 2 h after intravenous injection. (**H**) Representative PET/CT images 2 h after intravenous co-injection of ^68^Ga-DOTA-A1K2 or.^68^Ga-DOTA-A1K4 with gelofusin. (n = 6–7). (**I**) Ex vivo biodistribution profile, 2 h after intravenous injection. (**p < 0.01). (**J**) Tumor-to-blood, tumor-to-liver, and tumor-to-kidney ratios 2 h after injection. (**p < 0.01)

Incorporating a TEV protease recognition site allowed for the removal of the His-tag, reducing polarity and leaving only a single glycine residue at the C-terminus (Supplementary Fig. 2A-C). A1K1-His and A1K3-His were excluded due to their less favorable biodistribution profiles and radiolabeling stability, respectively. A1K2 and A1K4 were produced with a TEV sequence for His-tag removal (Supplementary Fig. 2D). Production yields were lower using this approach due to the addition of cleavage and purification steps. Affinities toward hMSLN remained unchanged (Table 1 and Fig. 3A).

Following CD analysis, 60 °C was confirmed as suitable for their radiolabeling. Conjugation efficiencies were satisfactory, with 0.69 ± 0.13 DOTA per sdAb for A1K2 and 0.72 ± 0.03 for A1K4. Radiolabelling, determined prior to purification, yielded high initial RCPs (A1K2: 95.0 ± 2.0%; A1K4: 94.3 ± 2.1%) and maintained stability after 2 h (A1K2: 92.7 ± 1.5%; A1K4: 93.0 ± 1.0%). After purification the purity was > 95%, with only minor impurities (< 1%) such as a peak observed at ~ 6 min (Table 1 and Fig. 3 B-D).

### In vivo specificity

For the displacement assay, PET imaging was acquired prior and after injection of a 100-fold excess competitor A1-His or irrelevant sdAb R3B23-His (Fig. 3E). ^68^Ga -DOTA-A1K4 displacement resulted in a marked reduction in tumor intensity, while no change was observed in other organs. Biodistribution, determined by gamma well counting of tissue samples, confirmed the 55% reduction in tumor activity, resulting in reduced tumor-to-blood and tumor-to-kidney ratios (Fig. 3G and STable 4–6).

Moreover, in vivo competition study using co-injection of a 100-fold excess competitor or irrelevant sdAb further confirmed the specificity of tumor uptake, as illustrated by a significant 43% reduction in tumor uptake (*p* < 0.01). (SFig. 3 A-C and STable 7–9).

### Comparative in vivo analysis of ^68^Ga-DOTA-A1K4 Vs.^68^Ga-DOTA-A1K2

First, DOTA-A1K4 was used to confirm that previously published methods can be employed to reduce kidney retention. Removal of the hexahistidine tag and co-injection with gelofusin significantly decrease kidney uptake by 78% and 75%, respectively (*p* < 0.05). Furthermore, combining both strategies further reduced renal retention by 86% (*p* < 0.0001), without affecting tumor uptake. As a result, tumor-to-kidney ratio was improved it by a factor of 6.7. Interestingly, His-tag removal alone also decreased hepatic retention by 61% and spleen retention by 68% (*p* < 0.01). (SFig. 4 A-C and STable 10–12).

His-tag removal and coinjection with gelofusin were therefore implemented for the in vivo comparison of the lead candidates ^68^Ga-DOTA-A1K4 and ^68^Ga-DOTA-A1K2. PET/CT imaging acquired 1 h after the injection of 9 MBq these sdAb showed no significant visual differences between groups (Fig. 3H). However, biodistribution analysis revealed that kidney retention was significantly 59% lower for ^68^Ga -DOTA-A1K2, compare to ^68^Ga -DOTA-A1K4 (*p* < 0.01) (Fig. 3I). This more than twofold reduction in renal uptake led to notable improvements in the tumor-to-kidney ratio for A1K2 (*p* < 0.01), and it also improves the tumor-to-liver ratio (*p* < 0.01) (Fig. 3J and STable 13–15).

## Discussion

Site-specific labeling of peptides and proteins plays a critical role in improving the precision and effectiveness of radiolabeled agents for theranostic purposes. A notable advancement in this field was the successful site-specific labeling of 18F-FDG on proteins, a breakthrough that significantly enhanced diagnostic imaging capabilities (Wuest et al. 2009; Namavari et al. 2009). Modern approaches employ a variety of innovative techniques to achieve accurate and selective labelling. These strategies are highly efficient, selective, and compatible with physiological conditions. One widely used method involves the incorporation of non-natural amino acids (nnAAs) into peptides or proteins, enabling bioorthogonal conjugation reactions such as strain-promoted azide-alkyne cycloaddition (SPAAC) and inverse electron demand Diels–Alder (IEDDA) reactions (Lee 2019). CoLDR chemistry (Covalent Labeling via Directed Reaction) uses short peptides that specifically interact with electrophilic groups to achieve precise covalent labeling (Reddi et al. 2021). Genetic encoding provides another effective strategy, allowing the incorporation of nnAAs with reactive functionalities into proteins through engineered tRNA-synthetase systems (Ma et al. 2024). Affinity-directed labeling exploits the specific binding of ligands to target proteins, enabling the precise positioning of labeling reagents without the need for genetic modification (Kim et al. 2024). Peptide-tag-based methods, including systems like SNAP-tag, CLIP-tag, and HaloTag, facilitate site-specific conjugation via chemical or enzymatic reactions (Lotze et al. 2016). Enzyme-based approaches, such as those utilizing transglutaminases, sortases, or tyrosinases, rely on the inherent substrate specificity of enzymes to catalyze covalent modifications at defined protein sites (Sueda 2020).

These diverse techniques provide tailored solutions for improving the in vivo stability, pharmacokinetic properties, and targeting accuracy of radiolabeled agents while minimizing off-target effects. Site-specific labeling ensures that theranostic agents maintain their biological functionality and achieve precise localization of radioisotopes, making them indispensable for advanced imaging and radionuclide therapy. The integration of chemical and genetic innovations continues to propel progress in theranostics, fostering the development of highly specific and effective tools for personalized medicine.

In this proof of concept study, molecular engineering of the sdAb A1 directed against mesothelin was performed, successfully allowing straightforward site-specific radiolabeling. By selectively substituting three out of four lysine residues with arginine, A1-mutants containing a single reactive site for p-SCN-Bn-DOTA conjugation were generated and further evaluated in vitro and in vivo.

In silico simulation predicted that none of the four lysine residues contained within the framework of A1 were contacting the epitope region of the mesothelin protein. Moreover, they were found to be accessible and distant from contacting regions that mainly involved the CDR1 and CDR3, but also antiparallel beta sheets located within the framework downstream of the CDR2. In accordance with these predictions, no change in affinity were observed using SPR analysis. Due to the presence of a single lysine per sdAb, DOTA coupling was performed using a 50-fold molar excess of p-SCN-Bn-DOTA, that is higher than that previously employed by others (Puttemans et al. 2020). This allowed successful coupling for all 4 mutants. Thermal stability was also found to remained unchanged, thereby enabling radiolabelling at 60 °C, resulting in RCP > 95%. The mutant A1K3-His was found to present suboptimal stability at 2 h in the radiolabelling medium. Further studies will be conducted to determine if the same occurs in the injection buffer. In vivo in mice bearing human HCC70 tumors, modest differences were observed in between the 4 mutants biodistribution. However, the liver was readily visible by PET using A1K1-His and this finding was confirmed by significantly decreased tumor-to-blood and tumor-to-liver ratios. Consequently, A1K3 and A1K1 mutants were no longer considered and A1K2 and A1K4 were selected as the lead compounds. In accordance with previous studies, His-tag removal and co-injection with gelofusin significantly reduced their unspecific kidney retention (D’Huyvetter et al. 2014), while not modifying tumor uptake, that was demonstrated to be specific using in vivo competition. The fact that in vivo displacement experiment also successfully inhibited tumor retention confirmed the specificity of the tumor uptake and suggest that at least 50% of the sdAb remained un-internalized at 1 h. DOTA are known to provide negative charges, which could potentially decrease kidney reuptake (Baudhuin et al. 2021; Raheem et al. 2023). However, in this study, no statistical differences were observed between A1 that contained 1.8 DOTA and the mutants that contained approximately 0.7 DOTA, indicating that reducing the number of DOTA was not responsible for an increased dosimetry to this organ. This parameter should however be considered if a different chelator is employed.

Mutants of sdAb A1 enabling simple site-specific conjugation were therefore successfully produced and evaluated. A1K2 and A1K4 were found to be the best candidates for imaging mesothelin positive tumors. Based on this finding, an A1 derivative containing both K2 and K4 could be produce to improve the specific activity while avoiding the lower stability or liver retention observed with K1 and K3. However, since our final objective is to generate a therapeutic sdAb, A1K2 appears to be the most promising candidate due to its significantly lower kidney retention and therefore higher tumor-to-kidney ratio than that of A1K4.

Importantly, this study was conducted as a proof of concept, and most findings could have broader implications for the use of sdAb-based radiotracers in nuclear medicine (Wei et al. 2022; Bridoux et al. 2020). As a matter of fact, it is a simple method allowing direct site-specific coupling of bi-specific chelators readily following the bioproduction. In comparison to other methods, such as sortase coupling (Basuli et al. 2024; Morgan et al. 2022) or via a cysteine introduced at the C-terminal end. (Massa et al. 2014), it does not require any additional step, thereby leading to higher production yield as well as to an improvement in time and cost effectiveness. The absence of residual enzyme can also improve safety and ease the clinical transfer. This approach for site specific labelling is therefore simple and offers advantages over previously described generic methods. However, if a lysine is involved in contacting the epitope, it requires evaluating the most appropriate mutation in order not to compromise its affinity.

Furthermore, the influence of DOTA chelator position already showed its impact on affibody biodistribution and targeting properties (Perols et al. 2012), here we confirmed that the position of the DOTA chelator impact also for sdAbs. Therefore, choosing the optimal position for coupling DOTA could be benefitial for optimal dosimetry and theranostic application. In addition, selecting the most appropriate lysine residue for site specific labeling can also enable improving key parameters such as production yield, radiochemical stability or biodistribution. Even though differences between mutants can be modest, as illustrated in the present study by in vivo biodistribution evaluation, selecting the most potent one might improve parameters such as the dosimetry, that is of tremendous importance for theranostic applications. Production yield, while not essential for research purpose, can also be of great importance when moving to the clinic.

Theoretically, this method for site specific labeling could also be employed for proteins other than sdAb. However, it could be noted that sdAb (VHH) scaffold is particularly well conserved. Indeed the 4 lysines present in the scaffold of the sdAb A1 are found in most sdAb, thereby supporting the fact that this method can be considered for this particular class of theranostic agents, while for example scaffold of the variable regions of the heavy chain (VH) of conventional antibodies do not demonstrate such high a redundancy in lysine positions (Hirao et al. 2023; Mitchell and Colwell 2018).

## Conclusion

In this proof of concept study, we demonstrated that site specific coupling of bifunctional chelator on a sdAb side chain lysine can be performed using a simple approach relying on site specific mutations. Interestingly, selecting the lysine position to be conserved allowed improving key parameters for the development of radiopharmaceuticals, such as the radiochemical purity or the biodistribution profile. Such generic approach could be employed for most sdAbs, but also potentially to other protein-based radiotracers.

## Supplementary Information

Additional file 1.

## Data Availability

The datasets generated and analysed during the current study are available from the corresponding author on reasonable request.

## Abbreviations

%ID/g: Percent injected dose per gram
^177^Lu: 177-Lutétium
^68^Ga: 68-Gallium
CD: Circular dichroism
CT: Computed tomography
DOTA: 1,4,7,10-Tetraazacyclododecane-1,4,7,10-tetraacetic acid
HER2: Human epidermal growth factor receptor 2
HPLC: High-pressure liquid chromatography
IMAC: Immobilized metal affinity chromatography
MALDI-TOF: Matrix assisted laser desorption ionization time of flight
MMR: Macrophage mannose receptor
MSLN: Mesothelin
NOTA: 2,2′,2″-(1,4,7-Triazacyclononane-1,4,7-triyl)triacetic acid
PBS: Phosphate buffer saline
PET: Positron emission tomography
pLDDT: Predicted local distance difference test
RCP: Radiochemical purities
sdAb: Single domain antibody
SEC: Size exclusion chromatography
SPECT: Single photon emission computed tomography
SPR: Surface plasmon resonance
TEV: Tobacco etch virus
TNBC: Triple negative breast cancer
VHH: Variable regions of the heavy chain of heavy-chain-only antibodies
