# Supplementary material for: Enhancing theranostic potential of anti-mesothelin sdAb through site-specific labeling at a unique conserved lysine by molecular engineering
Source: EJNMMI Radiopharm Chem. 2025 Apr 28;10:19. doi: 10.1186/s41181-025-00340-z (PMC12037457; doi:10.1186/s41181-025-00340-z)
Supplement: Supplementary file 1 — Additional file 1. [file 41181_2025_340_MOESM1_ESM.docx]

***Enhancing theranostic potential of anti-mesothelin sdAb through site-specific labeling at a unique conserved lysine by molecular engineering***

**Author names and affiliations**

*Émilien N’Guessan^1^, Florian Raes^1^, Mitra Ahmadi^1^, Sandrine Bacot^1^, Laurent Dumas^1^, Julien Leenhardt^1, 2^, Marlène Debiossat^1^, Clémence André^1^, Jean-Luc Lenormand^3^, Catherine Ghezzi^1^, Daniel Fagret^1^, Charlotte Lombardi ^1, 3^*^#^*, Alexis Broisat^1^*^#^ *^1^ Univ. Grenoble Alpes, INSERM U1039, LRB, Grenoble, France*

*^2^ Univ. Grenoble Alpes, CHU Grenoble Alpes, Department of Nuclear Medicine, Grenoble, France*

*^3^ Univ. Grenoble Alpes, CNRS U5525, TIMC-Tree, La Tronche, France*

# Contributed equally to this work

***Supplementary figures***


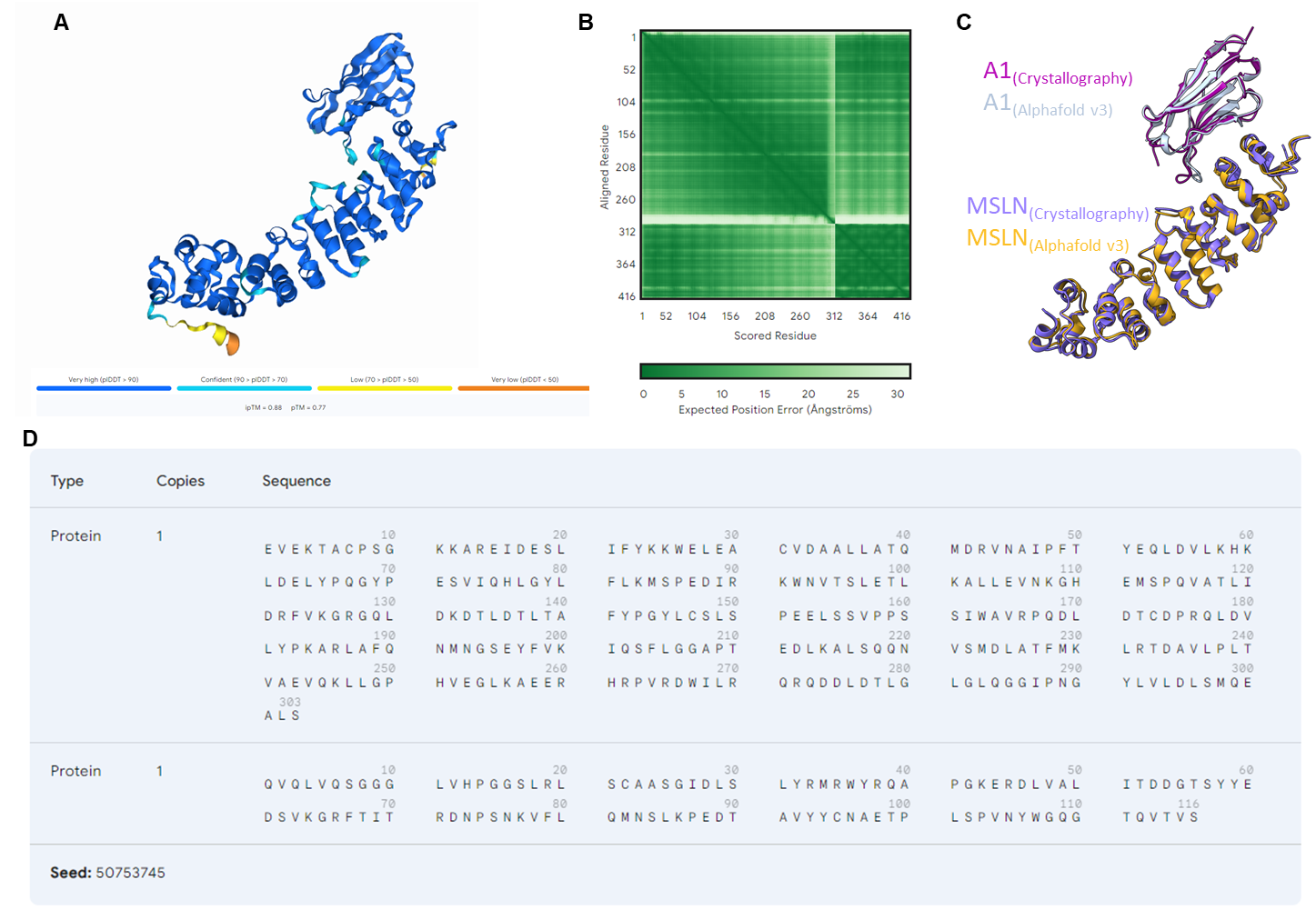


**Supplementary Figure 1 |** Predicted secondary structures of the complex sdAb A1 with mesothelin obtained on AlphaFold v3

1. Predictive confidence of the secondary structures of the complex sdAb A1 with mesothelin obtained on AlphaFold v3
2. Predicted aligned error (PAE) of the secondary structures of the complex sdAb A1 with mesothelin obtained on AlphaFold v3
3. Superposed cartoon representations of the secondary structures of sdAb A1 obtained by crystallography (purple) or by Alpha Fold v3 (grey), and of mesothelin obtained by crystallography (violet) or by Alpha Fold v3 (gold).
4. Sequence and seed use on AlphaFold v3. First sequence corresponds to mesothelin protein and the second one to sdAb A1

|  | **^68^Ga-DOTA-A1-His** | | | **^68^Ga-DOTA-A1K1-His** | | | **^68^Ga-DOTA-A1K2-His** | | | **^68^Ga-DOTA-A1K3-His** | | | **^68^Ga-DOTA-A1K4-His** | | |
| --- | --- | --- | --- | --- | --- | --- | --- | --- | --- | --- | --- | --- | --- | --- | --- |
|  | Mean | ± | SD | Mean | ± | SD | Mean | ± | SD | Mean | ± | SD | Mean | ± | SD |
| ID t inj (MBq) | 3.58 | ± | 0.86 | 2.49* | ± | 0.78 | 3.87 | ± | 0.68 | 3.70 | ± | 0.18 | 4.61 | ± | 0.86 |
| Weight mice (g) | 17.30 | ± | 6.51 | 19.46 | ± | 1.94 | 20.22 | ± | 1.54 | 19.95 | ± | 0.56 | 20.23 | ± | 1.26 |
| Tumor size (mm^3^) | 207.35 | ± | 77.56 | 221.28 | ± | 93.60 | 222.34 | ± | 85.95 | 214.52 | ± | 81.67 | 186.47 | ± | 78.78 |

**Table 1.** Details of the different groups of mice injected in the *in vivo* comparisons of the sdAb ^68^Ga-DOTA-A1-His, with its 4 mutants ^68^Ga-DOTA-A1K1-His, ^68^Ga-DOTA-A1K2-His, ^68^Ga-DOTA-A1K3-His and ^68^Ga-DOTA-A1K4-His. Expressed as mean ± SD (standard deviation (n=9, except ^68^Ga-DOTA-A1K4-His n=6). *****Significantly different from control sdAb ^68^Ga-DOTA-A1-His (p < 0.05).

|  | **^68^Ga-DOTA-A1-His** | | | **^68^Ga-DOTA-A1K1-His** | | | **^68^Ga-DOTA-A1K2-His** | | | **^68^Ga-DOTA-A1K3-His** | | | **^68^Ga-DOTA-A1K4-His** | | |
| --- | --- | --- | --- | --- | --- | --- | --- | --- | --- | --- | --- | --- | --- | --- | --- |
| Organs | Mean | ± | SD | Mean | ± | SD | Mean | ± | SD | Mean | ± | SD | Mean | ± | SD |
| Blood | 0.79 | ± | 0.22 | 1.11 | ± | 0.23 | 0.57 | ± | 0.10 | 0.54* | ± | 0.06 | 0.55 | ± | 0.13 |
| Bone | 0.49 | ± | 0.16 | 0.63 | ± | 0.11 | 0.31* | ± | 0.06 | 0.29** | ± | 0.04 | 0.41 | ± | 0.14 |
| Brain | 0.03 | ± | 0.01 | 0.05 | ± | 0.02 | 0.02 | ± | 0.01 | 0.02* | ± | 0.00 | 0.02 | ± | 0.01 |
| Brown fat | 0.39 | ± | 0.08 | 0.44 | ± | 0.19 | 0.44 | ± | 0.47 | 0.24** | ± | 0.06 | 0.31 | ± | 0.12 |
| Genital tract | 0.98 | ± | 0.43 | 1.08 | ± | 0.26 | 0.95 | ± | 0.54 | 0.78 | ± | 0.22 | 0.70 | ± | 0.21 |
| Heart | 0.34 | ± | 0.13 | 0.48 | ± | 0.15 | 0.28 | ± | 0.08 | 0.29 | ± | 0.06 | 0.30 | ± | 0.06 |
| Intestine | 0.56 | ± | 0.21 | 0.60 | ± | 0.20 | 0.44 | ± | 0.09 | 0.49 | ± | 0.11 | 0.54 | ± | 0.12 |
| **Kidney** | **123.25** | **±** | **22.03** | **101.18*** | **±** | **21.77** | **111.38** | **±** | **24.23** | **94.44**** | **±** | **5.39** | **100.94** | **±** | **13.17** |
| Liver | 1.11 | ± | 0.43 | 2.20 | ± | 1.30 | 0.76 | ± | 0.41 | 0.57* | ± | 0.29 | 1.02 | ± | 0.63 |
| Lung | 1.00 | ± | 0.60 | 1.14** | ± | 0.24 | 0.84 | ± | 0.12 | 0.95 | ± | 0.19 | 1.00 | ± | 0.17 |
| Lymph nodes | 0.80 | ± | 0.58 | 0.95 | ± | 0.73 | 0.63 | ± | 0.30 | 0.44 | ± | 0.16 | 0.57 | ± | 0.19 |
| Muscle | 0.14 | ± | 0.02 | 0.16 | ± | 0.03 | 0.17 | ± | 0.13 | 0.12 | ± | 0.03 | 0.19 | ± | 0.05 |
| Ovaries | 0.82 | ± | 0.27 | 0.86 | ± | 0.30 | 0.72 | ± | 0.20 | 0.58* | ± | 0.12 | 0.76 | ± | 0.21 |
| Pancreas | 0.33 | ± | 0.05 | 0.41 | ± | 0.09 | 0.24** | ± | 0.05 | 0.28 | ± | 0.05 | 0.32 | ± | 0.07 |
| Salivary glands | 0.36 | ± | 0.05 | 0.42 | ± | 0.10 | 0.28 | ± | 0.05 | 0.24** | ± | 0.05 | 0.31 | ± | 0.10 |
| Skin | 0.60 | ± | 0.08 | 0.62 | ± | 0.15 | 0.44** | ± | 0.08 | 0.43** | ± | 0.05 | 0.55 | ± | 0.20 |
| Spleen | 0.80 | ± | 0.34 | 1.76* | ± | 0.77 | 0.56 | ± | 0.27 | 0.49* | ± | 0.24 | 0.63 | ± | 0.27 |
| Stomach | 0.44 | ± | 0.15 | 0.43 | ± | 0.10 | 0.39 | ± | 0.23 | 0.33 | ± | 0.11 | 0.42 | ± | 0.10 |
| **Tumor HCC70** | **2.69** | **±** | **0.74** | **2.12** | **±** | **0.52** | **1.62***** | **±** | **0.35** | **1.78**** | **±** | **0.46** | **2.51** | **±** | **0.86** |
| White fat | 0.29 | ± | 0.06 | 0.39 | ± | 0.25 | 0.27 | ± | 0.09 | 0.30 | ± | 0.10 | 0.35 | ± | 0.09 |

**Table 2.** *Ex vivo* biodistribution datas 2 hours after intravenous injection of the sdAb ^68^Ga-DOTA-A1-His, or one of its 4 mutants ^68^Ga-DOTA-A1K1-His, ^68^Ga-DOTA-A1K2-His, ^68^Ga-DOTA-A1K3-His and ^68^Ga-DOTA-A1K4-His, in *Athymic Nude* mice bearing HCC70 xenografts. Expressed as mean of % ID/g tissue ± SD (standard deviation); (n=9 except ^68^Ga-DOTA-A1K4-His n=6). *****Significantly different from control sdAb ^68^Ga-DOTA-A1-His (*=p < 0.05 ; **= p < 0.01 ; ***= p < 0.001).

|  | **^68^Ga-DOTA-A1-His** | | | **^68^Ga-DOTA-A1K1-His** | | | **^68^Ga-DOTA-A1K2-His** | | | **^68^Ga-DOTA-A1K3-His** | | | **^68^Ga-DOTA-A1K4-His** | | |
| --- | --- | --- | --- | --- | --- | --- | --- | --- | --- | --- | --- | --- | --- | --- | --- |
|  | Mean | ± | SD | Mean | ± | SD | Mean | ± | SD | Mean | ± | SD | Mean | ± | SD |
| Tumor/Blood | 3.66 | ± | 1.35 | 1.99* | ± | 0.67 | 2.86 | ± | 0.41 | 3.26 | ± | 0.70 | 4.49 | ± | 0.62 |
| Tumor/Liver | 2.69 | ± | 1.20 | 1.32* | ± | 0.97 | 2.48 | ± | 0.78 | 3.43 | ± | 0.80 | 3.00 | ± | 1.08 |
| Tumor/Muscle | 19.18 | ± | 5.12 | 13.88* | ± | 3.02 | 14.21* | ± | 5.98 | 14.96 | ± | 2.66 | 14.98 | ± | 5.28 |
| Tumor/Kidney | 0.02 | ± | 0.01 | 0.02 | ± | 0.01 | 0.02* | ± | 0.01 | 0.02 | ± | 0.00 | 0.03 | ± | 0.01 |

**Table 3.** Tumor-to-organ ratios of %ID/g values 2h after intravenous injection of the sdAb ^68^Ga-DOTA-A1-His, or one of its 4 mutants ^68^Ga-DOTA-A1K1-His, ^68^Ga-DOTA-A1K2-His, ^68^Ga-DOTA-A1K3-His and ^68^Ga-DOTA-A1K4-His, in *Athymic Nude* mice bearing HCC70 xenografts. Expressed as mean ± SD (standard deviation) (n=9 except ^68^Ga-DOTA-A1K4-His n=6). *****Significantly different from control sdAb ^68^Ga-DOTA-A1-His (*=p < 0.05).

**
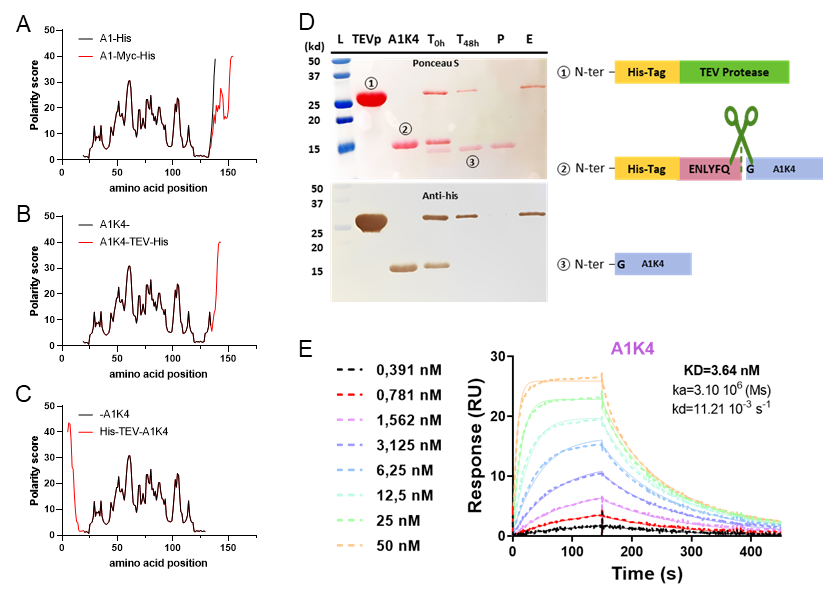
Supplementary Figure 2 | Methodology of the enzymatic cleavage of the His-Tag by the TEV protease**

1. Zimmerman Plot - polarity score of A1-His-Myc and A1-His
2. Zimmerman Plot - polarity score of A1K4 with TEV protease cleavage site in C-ter, in red the cleaved part
3. Zimmerman Plot - polarity score of A1K4 with TEV protease cleavage site N-ter, in red the cleaved part
4. S Ponceau membrane and Western Blot anti-His depicting the deletion of the his-tag from sdAb A1K4. L: Ladder; TEVp: TEV protease; A1K4: sdAb His-TEV-A1K4; T_0h_: TEVp and His-TEV-A1K4; T_48h_: TEVp and A1K4 after 48h enzymatic cleavage; P: A1K4 after IMAC purification; E: Elution of IMAC after purification

|  | **^68^Ga-DOTA-A1K4**  **+ 100x sdAbs irrelevant** | | | **^68^Ga-DOTA-A1K4**  **+ 100x sdAbs A1-His** | | |
| --- | --- | --- | --- | --- | --- | --- |
|  | Mean | ± | SD | Mean | ± | SD |
| Inject dose (MBq) | 9.60 | ± | 0.60 | 9.11 | ± | 0.73 |
| Weight mice (g) | 18.23 | ± | 1.66 | 19.33 | ± | 0.36 |
| Tumor size (mm^3^) | 145.24 | ± | 28.51 | 164.87 | ± | 47.76 |

**Table 4.** Details of the two groups of mice injected in the *in vivo* displacement study of the sdAb ^68^Ga-DOTA-A1K4. Expressed as mean ± SD (standard deviation (n=3)

|  | **^68^Ga-DOTA-A1K4**  **+ 100x sdAbs irrelevant** | | | **^68^Ga-DOTA-A1K4**  **+ 100x sdAbs A1-His** | | |
| --- | --- | --- | --- | --- | --- | --- |
| Organs | Mean | ± | SD | Mean | ± | SD |
| Blood | 0.45 | ± | 0.16 | 0.57 | ± | 0.05 |
| Bone | 0.51 | ± | 0.18 | 0.33 | ± | 0.06 |
| Brain | 0.02 | ± | 0.01 | 0.02 | ± | 0.01 |
| Brown fat | 0.19 | ± | 0.05 | 0.29 | ± | 0.09 |
| Genital tract | 0.79 | ± | 0.41 | 0.64 | ± | 0.10 |
| Heart | 0.17 | ± | 0.03 | 0.24 | ± | 0.07 |
| Intestine | 0.31 | ± | 0.07 | 0.30 | ± | 0.09 |
| Kidney | 19.82 | ± | 2.61 | 20.37 | ± | 1.71 |
| Liver | 0.26 | ± | 0.06 | 0.29 | ± | 0.03 |
| Lung | 0.76 | ± | 0.09 | 0.57 | ± | 0.25 |
| Lymph nodes | 0.34 | ± | 0.15 | 0.38 | ± | 0.13 |
| Muscle | 0.25 | ± | 0.24 | 0.13 | ± | 0.05 |
| Ovaries | 0.47 | ± | 0.11 | 0.44 | ± | 0.10 |
| Pancreas | 0.35 | ± | 0.21 | 0.19 | ± | 0.03 |
| Salivary glands | 0.22 | ± | 0.04 | 0.22 | ± | 0.03 |
| Skin | 0.37 | ± | 0.07 | 0.48 | ± | 0.11 |
| Spleen | 0.28 | ± | 0.00 | 0.25 | ± | 0.02 |
| Stomach | 0.14 | ± | 0.12 | 0.22 | ± | 0.12 |
| **Tumor HCC70** | **2.12** | **±** | **0.49** | **0.94** | **±** | **0.20** |
| White fat | 0.33 | ± | 0.12 | 0.19 | ± | 0.04 |

**Table 5.** *Ex vivo* biodistribution datas 2 hours after intravenous injection of ^68^Ga-DOTA-A1K4 and 1h after injection of 100x irrelevant sdab or 100x sdAb A1-His in Athymic Nude mice bearing HCC70 xenografts. Expressed as mean of % ID/g tissue ± SD (standard deviation); (n=3)

|  | **^68^Ga-DOTA-A1K4**  **+ 100x sdAbs irrelevant** | | | **^68^Ga-DOTA-A1K4**  **+ 100x sdAbs A1-His** | | |
| --- | --- | --- | --- | --- | --- | --- |
|  | Mean | ± | SD | Mean | ± | SD |
| Tumor/Blood | 4.83 | ± | 0.84 | 1.67 | ± | 0.35 |
| Tumor/Liver | 8.23 | ± | 0.91 | 3.18 | ± | 0.41 |
| Tumor/Muscle | 13.38 | ± | 8.39 | 7.61 | ± | 1.66 |
| Tumor/Kidney | 0.11 | ± | 0.03 | 0.05 | ± | 0.01 |

**Table 6.** Tumor-to-organ ratios of %ID/g values 2 hours after intravenous injection of ^68^Ga-DOTA-A1K4 and 1h after injection of 100x irrelevant sdab or 100x sdAb A-His1 in Athymic Nude mice bearing HCC70 xenografts. Expressed as mean ± SD (standard deviation) (n=3).

***
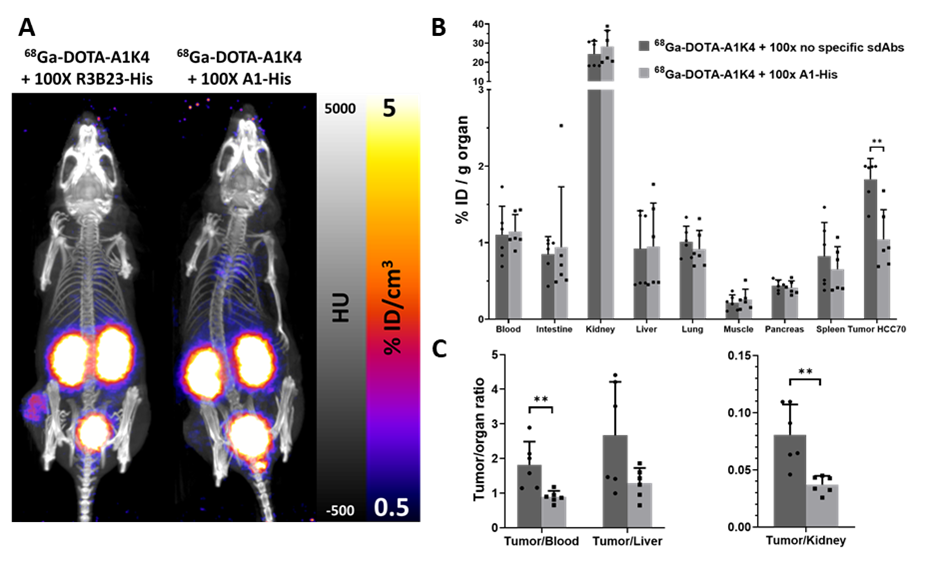
In vivo* competition study with ^68^Ga-DOTA-A1K4**

**Supplementary Figure 4 | Competition biodistribution study of ^68^Ga-DOTA-A1K4 in competition with A1-His in *Athymic Nude* mice bearing HCC70 xenografts (n=6).**

1. Representative images 1 hour after intravenous injection. Maximum intensity projection representative of merged PET/CT.
2. *Ex vivo* biodistribution profile 2 hours after intravenous injection.
3. *Ex vivo* biodistribution profile 2 hours after intravenous injection of tumor/blood, tumor/liver and tumor/kidney ratios.

|  | **^68^Ga-DOTA-A1K4-His**  **+ 100x sdAbs irrelevant** | | | **^68^Ga-DOTA-A1K4-His**  **+ 100x sdAbs A1-His** | | |
| --- | --- | --- | --- | --- | --- | --- |
|  | Mean | ± | SD | Mean | ± | SD |
| ID t cpt (MBq) | 0.22 | ± | 0.20 | 0.20 | ± | 0.18 |
| ID t inj (MBq) | 2.71 | ± | 1.46 | 2.28 | ± | 0.33 |
| Weight mice (g) | 18.60 | ± | 1.53 | 18.61 | ± | 0.93 |
| Tumor size (mm^3^) | 224.68 | ± | 93.35 | 218.65 | ± | 61.09 |

**Table 7.** Details of the two groups of mice injected in the *in vivo* competition study of the sdAb ^68^Ga-DOTA-A1K4. Expressed as mean ± SD (standard deviation (n=6)

|  | **^68^Ga-DOTA-A1K4-His**  **+ 100x sdAbs irrelevant** | | | **^68^Ga-DOTA-A1K4-His**  **+ 100x sdAbs A1-His** | | |
| --- | --- | --- | --- | --- | --- | --- |
| Organs | Mean | ± | SD | Mean | ± | SD |
| Blood | 1.10 | ± | 0.37 | 1.15 | ± | 0.22 |
| Bone | 0.98 | ± | 0.43 | 0.97 | ± | 0.24 |
| Brain | 0.03 | ± | 0.02 | 0.04 | ± | 0.01 |
| Brown fat | 0.54 | ± | 0.22 | 0.57 | ± | 0.20 |
| Genital tract | 1.02 | ± | 0.31 | 1.41 | ± | 0.74 |
| Heart | 0.40 | ± | 0.13 | 0.42 | ± | 0.14 |
| Intestine | 0.85 | ± | 0.23 | 0.94 | ± | 0.79 |
| Kidney | 24.40 | ± | 6.74 | 28.30 | ± | 8.40 |
| Liver | 0.92 | ± | 0.50 | 0.95 | ± | 0.57 |
| Lung | 1.01 | ± | 0.21 | 0.92 | ± | 0.24 |
| Lymph nodes | 0.73 | ± | 0.24 | 0.79 | ± | 0.50 |
| Muscle | 0.22 | ± | 0.10 | 0.26 | ± | 0.14 |
| Ovaries | 0.79 | ± | 0.23 | 0.76 | ± | 0.24 |
| Pancreas | 0.44 | ± | 0.07 | 0.41 | ± | 0.09 |
| Salivary glands | 0.44 | ± | 0.12 | 0.43 | ± | 0.13 |
| Skin | 0.61 | ± | 0.12 | 0.63 | ± | 0.27 |
| Spleen | 0.82 | ± | 0.44 | 0.65 | ± | 0.30 |
| Stomach | 0.59 | ± | 0.21 | 0.51 | ± | 0.22 |
| **Tumor HCC70** | **1.83** | **±** | **0.27** | **1.04**** | **±** | **0.39** |
| White fat | 0.41 | ± | 0.13 | 0.46 | ± | 0.40 |

**Table 8.** *Ex vivo* biodistribution datas 2 hours after intravenous injection of 68Ga-DOTA-A1K4 and 100x irrelevant sdab or 100x sdAb A1-His in Athymic Nude mice bearing HCC70 xenografts. Expressed as mean of % ID/g tissue ± SD (standard deviation); (n=6). *****Significantly different from control sdAb ^68^Ga-DOTA-A1K4-His + 100x Nb irrelevant (**= p < 0.01).

|  | **^68^Ga-DOTA-A1K4-His**  **+ 100x sdAbs irrelevant** | | | **^68^Ga-DOTA-A1K4-His**  **+ 100x sdAbs A1-His** | | |
| --- | --- | --- | --- | --- | --- | --- |
|  | Mean | ± | SD | Mean | ± | SD |
| Tumor/Blood | 1.82 | ± | 0.67 | 0.89** | ± | 0.17 |
| Tumor/Liver | 2.67 | ± | 1.55 | 1.29 | ± | 0.44 |
| Tumor/Muscle | 10.07 | ± | 4.95 | 4.33** | ± | 0.94 |
| Tumor/Kidney | 0.08 | ± | 0.03 | 0.04** | ± | 0.01 |

**Table 9.** Tumor-to-organ ratios of %ID/g values 2 hours after intravenous injection of 68Ga-DOTA-A1K4 and 100x irrelevant sdab or 100x sdAb A1-His in Athymic Nude mice bearing HCC70 xenografts. Expressed as mean ± SD (standard deviation) (n=6). *****Significantly different from control sdAb ^68^Ga-DOTA-A1K4-His + 100x Nb irrelevant (**= p < 0.01).

**In vivo kidney uptake reduction with ^68^Ga-DOTA-A1K4**

***
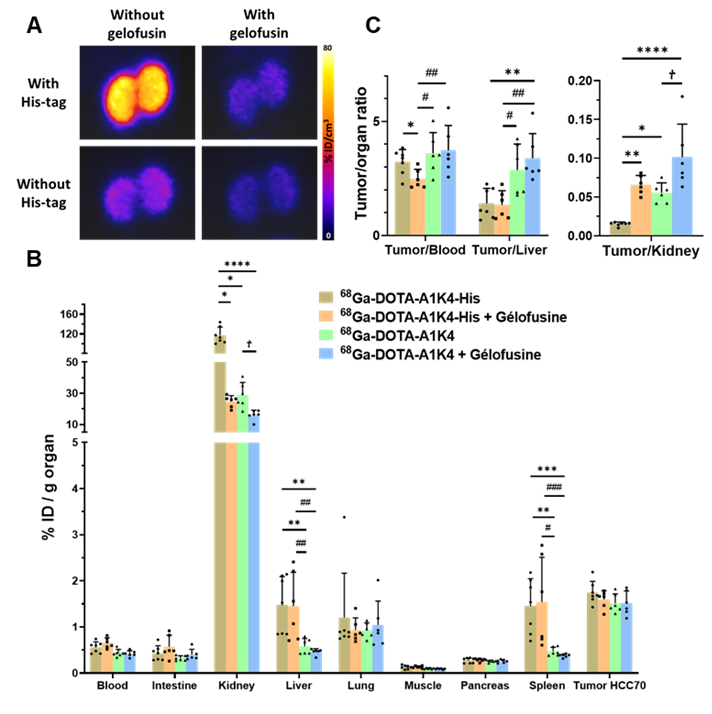
***

**Supplementary Figure 3 | Biodistribution study of ^68^Ga-DOTA-A1K4-His and ^68^Ga-DOTA-A1K4 ± co-injection Gelofusin in *Athymic Nude* mice bearing HCC70 xenografts** (**n=6-7).**

1. Maximum intensity projection representative of PET images.
2. *Ex vivo* biodistribution profile 2 hours after intravenous injection. *Significantly different from control sdAb ^68^Ga-DOTA-A1K4-His (*=p < 0.05 ; **= p < 0.01 ; ***= p < 0.001 ; ****=p<0.0001). ^#^Significantly different from sdAb ^68^Ga-DOTA-A1K4-His + gelofusin (^#^=p < 0.05 ; ^##^= p < 0.01 ; ^###^= p < 0.001). ^ⴕ^Significantly different from sdAb ^68^Ga-DOTA-A1K4 (^ⴕ^=p < 0.05).
3. *Ex vivo* biodistribution profile 2 hours after intravenous injection of tumor-to-blood, tumor-to-liver and tumor-to-kidney ratios.

|  | **^68^Ga-DOTA-A1K4-His (n=7)** | | | **^68^Ga-DOTA-A1K4-His**  **+ Gelofusin (n=6)** | | | **^68^Ga-DOTA-A1K4**  **(n=6)** | | | **^68^Ga-DOTA-A1K4**  **+ Gelofusin (n=6)** | | |
| --- | --- | --- | --- | --- | --- | --- | --- | --- | --- | --- | --- | --- |
|  | Mean | ± | SD | Mean | ± | SD | Mean | ± | SD | Mean | ± | SD |
| Inject dose (MBq) | 2.75 | ± | 0.18 | 1.95*** | ± | 0.16 | 2.52 | ± | 0.41 | 2.62 | ± | 0.15 |
| Weight mice (g) | 19.49 | ± | 0.97 | 19.52 | ± | 1.86 | 20.68 | ± | 1.76 | 19.95 | ± | 2.11 |
| Tumor size (mm^3^) | 359.17 | ± | 352.91 | 402.53 | ± | 225.50 | 449.05 | ± | 406.56 | 374.75 | ± | 169.62 |

**Table 10.** Details of the different groups of mice injected in the *in vivo* comparisons of the sdAb ^68^Ga-DOTA-A1K4-His, with ^68^Ga-DOTA-A1K4, ± gelofusin. Expressed as mean ± SD (standard deviation) (n=6-7). *****Significantly different from control sdAb ^68^Ga-DOTA-A1K4-His (***= p < 0.001).

|  | **^68^Ga-DOTA-A1K4-His** | | | **^68^Ga-DOTA-A1K4-His**  **+ Gelofusin** | | | **^68^Ga-DOTA-A1K4** | | | **^68^Ga-DOTA-A1K4**  **+ Gelofusin** | | |
| --- | --- | --- | --- | --- | --- | --- | --- | --- | --- | --- | --- | --- |
| Organs | Mean | ± | SD | Mean | ± | SD | Mean | ± | SD | Mean | ± | SD |
| Blood | 0.56 | ± | 0.12 | 0.65 | ± | 0.11 | 0.43^##^ | ± | 0.08 | 0.42*^##^ | ± | 0.07 |
| Bone | 0.40 | ± | 0.08 | 0.45 | ± | 0.08 | 0.34^#^ | ± | 0.08 | 0.30*^##^ | ± | 0.06 |
| Brain | 0.02 | ± | 0.00 | 0.03 | ± | 0.01 | 0.02^#^ | ± | 0.00 | 0.02^##^ | ± | 0.00 |
| Brown fat | 0.26 | ± | 0.04 | 0.29 | ± | 0.05 | 0.19*^##^ | ± | 0.03 | 0.20*^##^ | ± | 0.03 |
| Genital tract | 1.05 | ± | 0.77 | 0.88 | ± | 0.39 | 0.56^#^ | ± | 0.15 | 0.53^##^ | ± | 0.09 |
| Heart | 0.26 | ± | 0.04 | 0.28 | ± | 0.04 | 0.22*^#^ | ± | 0.04 | 0.21*^##^ | ± | 0.02 |
| Intestine | 0.43 | ± | 0.16 | 0.57 | ± | 0.25 | 0.32^#^ | ± | 0.05 | 0.40 | ± | 0.12 |
| Kidney | 117.09 | ± | 16.68 | 24.73* | ± | 3.76 | 28.72* | ± | 8.20 | 16.03****^ⴕ^ | ± | 3.22 |
| Liver | 1.47 | ± | 0.62 | 1.45 | ± | 0.74 | 0.58**^##^ | ± | 0.17 | 0.46**^##^ | ± | 0.07 |
| Lung | 1.20 | ± | 0.96 | 0.93 | ± | 0.26 | 0.92 | ± | 0.17 | 1.04 | ± | 0.52 |
| Lymph nodes | 0.51 | ± | 0.08 | 0.60 | ± | 0.17 | 0.46^#^ | ± | 0.19 | 0.43 | ± | 0.08 |
| Muscle | 0.13 | ± | 0.04 | 0.13 | ± | 0.02 | 0.10^#^ | ± | 0.01 | 0.09*^#^ | ± | 0.01 |
| Ovaries | 0.51 | ± | 0.20 | 0.77 | ± | 0.47 | 0.42 | ± | 0.14 | 0.43 | ± | 0.10 |
| Pancreas | 0.27 | ± | 0.06 | 0.28 | ± | 0.03 | 0.26 | ± | 0.02 | 0.26 | ± | 0.03 |
| Salivary glands | 0.25 | ± | 0.05 | 0.25 | ± | 0.04 | 0.20^#^ | ± | 0.03 | 0.20^#^ | ± | 0.03 |
| Skin | 0.58 | ± | 0.28 | 0.50 | ± | 0.07 | 0.48 | ± | 0.17 | 0.43 | ± | 0.14 |
| Spleen | 1.46 | ± | 0.59 | 1.54 | ± | 0.97 | 0.47**^#^ | ± | 0.09 | 0.38***^###^ | ± | 0.04 |
| Stomach | 0.26 | ± | 0.11 | 0.33* | ± | 0.05 | 0.28 | ± | 0.06 | 0.28 | ± | 0.09 |
| **Tumor HCC70** | **1.75** | **±** | **0.24** | **1.59** | **±** | **0.19** | **1.51** | **±** | **0.21** | **1.52** | **±** | **0.26** |
| White fat | 0.25 | ± | 0.09 | 0.30 | ± | 0.16 | 0.17^#^ | ± | 0.04 | 0.18 | ± | 0.05 |

**Table 11.** *Ex vivo* biodistribution datas 2 hours after intravenous injection of the sdAb ^68^Ga-DOTA-A1K4-His, or ^68^Ga-DOTA-A1K4, (± gelofusin) in *Athymic Nude* mice bearing HCC70 xenografts. Expressed as mean of % ID/g tissue ± SD (standard deviation); (n=6-7). *****Significantly different from control sdAb ^68^Ga-DOTA-A1K4-His (*=p < 0.05 ; **= p < 0.01 ; ***= p < 0.001 ; ****=p<0.0001). ^#^Significantly different from sdAb ^68^Ga-DOTA-A1K4-His + gelofusin (^#^=p < 0.05 ; ^##^= p < 0.01 ; ^###^= p < 0.001). ^ⴕ^Significantly different from sdAb ^68^Ga-DOTA-A1K4 (^ⴕ^=p < 0.05).

|  | **^68^Ga-DOTA-A1K4-His** | | | **^68^Ga-DOTA-A1K4-His**  **+ Gelofusin** | | | **^68^Ga-DOTA-A1K4** | | | **^68^Ga-DOTA-A1K4**  **+ Gelofusin** | | |
| --- | --- | --- | --- | --- | --- | --- | --- | --- | --- | --- | --- | --- |
|  | Mean | ± | SD | Mean | ± | SD | Mean | ± | SD | Mean | ± | SD |
| Tumor/Blood | 3.22 | ± | 0.55 | 2.48* | ± | 0.42 | 3.61^#^ | ± | 0.91 | 3.74^##^ | ± | 1.09 |
| Tumor/Liver | 1.41 | ± | 0.66 | 1.34 | ± | 0.62 | 2.87^#^ | ± | 1.14 | 3.38**^##^ | ± | 1.08 |
| Tumor/Muscle | 14.75 | ± | 4.22 | 12.40 | ± | 3.15 | 15.05 | ± | 1.79 | 16.56^#^ | ± | 2.19 |
| Tumor/Kidney | 0.02 | ± | 0.00 | 0.07** | ± | 0.01 | 0.06* | ± | 0.01 | 0.10****^ⴕ^ | ± | 0.04 |

**Table 12.** Tumor-to-organ ratios of %ID/g values 2h after intravenous injection of the sdAb ^68^Ga-DOTA-A1K4-His, or ^68^Ga-DOTA-A1K4, (± gelofusin) in *Athymic Nude* mice bearing HCC70 xenografts. Expressed as mean ± SD (standard deviation) (n=6-7). *****Significantly different from control sdAb ^68^Ga-DOTA-A1K4-His (*=p < 0.05 ; **= p < 0.01 ; ****=p<0.0001). ^#^Significantly different from sdAb ^68^Ga-DOTA-A1K4-His + gelofusin (^#^=p < 0.05 ; ^##^= p < 0.01). ^ⴕ^Significantly different from sdAb ^68^Ga-DOTA-A1K4 (^ⴕ^=p < 0.05).

|  | **^68^Ga-DOTA-A1K2 (n=7)** | | | **^68^Ga-DOTA-A1K4 (n=6)** | | |
| --- | --- | --- | --- | --- | --- | --- |
|  | Mean | ± | SD | Mean | ± | SD |
| Inject dose (MBq) | 2.93 | ± | 0.40 | 2.62* | ± | 0.15 |
| Weight mice (g) | 18.86 | ± | 1.45 | 19.95 | ± | 2.11 |
| Tumor size (mm^3^) | 192.18 | ± | 123.80 | 374.75* | ± | 169.62 |

**Table 13.** Details of the two groups of mice injected in the *in vivo* comparisons of the sdAb ^68^Ga-DOTA-A1K2, with ^68^Ga-DOTA-A1K2 (+gelofusin), in *Athymic Nude* mice bearing HCC70 xenografts. Expressed as mean ± SD (standard deviation (n=6-7). *****Significantly different from control sdAb ^68^Ga-DOTA-A1K2 (*=p < 0.05).

|  | **^68^Ga-DOTA-A1K2 (n=7)** | | | **^68^Ga-DOTA-A1K4 (n=6)** | | |
| --- | --- | --- | --- | --- | --- | --- |
| Organs | Mean | ± | SD | Mean | ± | SD |
| Blood | 0.34 | ± | 0.07 | 0.42 | ± | 0.07 |
| Bone | 0.26 | ± | 0.09 | 0.30 | ± | 0.06 |
| Brain | 0.01 | ± | 0.01 | 0.02 | ± | 0.00 |
| Brown fat | 0.15 | ± | 0.04 | 0.20* | ± | 0.03 |
| Genital tract | 0.44 | ± | 0.11 | 0.53 | ± | 0.09 |
| Heart | 0.15 | ± | 0.03 | 0.21* | ± | 0.02 |
| Intestine | 0.61 | ± | 0.31 | 0.40 | ± | 0.12 |
| Kidney | 6.51 | ± | 0.54 | 16.03** | ± | 3.22 |
| Liver | 0.25 | ± | 0.04 | 0.46** | ± | 0.07 |
| Lung | 0.51 | ± | 0.10 | 1.04** | ± | 0.52 |
| Lymph nodes | 0.33 | ± | 0.10 | 0.43 | ± | 0.08 |
| Muscle | 0.23 | ± | 0.23 | 0.09 | ± | 0.01 |
| Ovaries | 0.37 | ± | 0.18 | 0.43 | ± | 0.10 |
| Pancreas | 0.17 | ± | 0.03 | 0.26** | ± | 0.03 |
| Salivary glands | 0.17 | ± | 0.02 | 0.20 | ± | 0.03 |
| Skin | 0.46 | ± | 0.25 | 0.43 | ± | 0.14 |
| Spleen | 0.25 | ± | 0.08 | 0.38** | ± | 0.04 |
| Stomach | 0.23 | ± | 0.12 | 0.28 | ± | 0.09 |
| **Tumor HCC70** | **1.55** | **±** | **0.50** | **1.52** | **±** | **0.26** |
| White fat | 0.16 | ± | 0.03 | 0.18 | ± | 0.05 |

**Table 14.** *Ex vivo* biodistribution datas 2 hours after intravenous injection of the sdAb ^68^Ga-DOTA-A1K2, with ^68^Ga-DOTA-A1K2 (+gelofusin), in *Athymic Nude* mice bearing HCC70 xenografts. Expressed as mean of % ID/g tissue ± SD (standard deviation); (n=6-7). *****Significantly different from control sdAb ^68^Ga-DOTA-A1K2 (*=p < 0.05 ; **= p < 0.01).

|  | **^68^Ga-DOTA-A1K2 (n=7)** | | | **^68^Ga-DOTA-A1K4 (n=6)** | | |
| --- | --- | --- | --- | --- | --- | --- |
|  | Mean | ± | SD | Mean | ± | SD |
| Tumor/Blood | 4.76 | ± | 1.67 | 3.74 | ± | 1.09 |
| Tumor/Liver | 6.32 | ± | 1.83 | 3.38** | ± | 1.08 |
| Tumor/Muscle | 12.04 | ± | 7.03 | 16.56 | ± | 2.19 |
| Tumor/Kidney | 0.24 | ± | 0.10 | 0.10** | ± | 0.04 |

**Table 15.** Tumor-to-organ ratios of %ID/g values 2 hours after intravenous injection of the sdAb ^68^Ga-DOTA-A1K2, with ^68^Ga-DOTA-A1K2 (+gelofusin), in *Athymic Nude* mice bearing HCC70 xenografts. Expressed as mean ± SD (standard deviation) (n=6-7). *****Significantly different from control sdAb ^68^Ga-DOTA-A1K2 (**= p < 0.01).
